# Computational systems biology approaches to cellular aging—Integrating network maps and dynamical models

**DOI:** 10.1002/qub2.70007

**Published:** 2025-05-26

**Authors:** Hetian Su, Nan Hao

**Affiliations:** ^1^ Department of Molecular Biology School of Biological Sciences University of California San Diego La Jolla California USA; ^2^ Synthetic Biology Institute University of California San Diego La Jolla California USA; ^3^ Department of Bioengineering University of California San Diego La Jolla California USA

**Keywords:** cellular aging, gene regulatory network, mathematical model, nonlinear dynamics, systems biology

## Abstract

Cellular aging is a multifaceted complex process. Many genes and factors have been identified that regulate cellular aging. However, how these genes and factors interact with one another and how these interactions drive the aging processes in single cells remain largely unclear. Recently, computational systems biology has demonstrated its potential to empower aging research by providing quantitative descriptions and explanations of complex aging phenotypes, mechanistic insights into the emergent dynamic properties of regulatory networks, and testable predictions that can guide the design of new experiments and interventional strategies. In general, current complex systems approaches can be categorized into two types: (1) network maps that depict the topologies of large‐scale molecular networks without detailed characterization of the dynamics of individual components and (2) dynamical models that describe the temporal behavior in a particular set of interacting factors. In this review, we discuss examples that showcase the application of these approaches to cellular aging with a specific focus on the progress in quantifying and modeling the replicative aging of budding yeast *Saccharomyces cerevisiae*. We further propose potential strategies for integrating network maps and dynamical models toward a more comprehensive, mechanistic, and predictive understanding of cellular aging. Finally, we outline directions and questions in aging research where systems‐level approaches may be especially powerful.

## 1 INTRODUCTION

Aging is a defining factor in many diseases that have become increasingly significant global health burdens, including neurodegeneration, cancer, and diabetes [1]. As organismal aging is prohibitively sophisticated, much progress in understanding the basic mechanisms of aging comes from studies of aging processes in individual cells [2]. Many genes that regulate cellular lifespan and aging‐related functional declines have been characterized [3, 4, 5, 6, 7]. Based on the investigations of individual genes, proteins, and metabolites, previous studies have advanced our understanding of aging by delineating multiple major conserved aging pathways or processes [5, 8, 9, 10, 11]. Yet, understanding how these factors and pathways interact with one another and how these interactions operate collectively to drive the dynamics of aging process remains largely elusive. In addition, stochasticity is another important yet difficult‐to‐study factor that underlies the different rates and phenotypes of aging among individual cells. Traditional reductionist approaches are limited in addressing the totality of such complexity. In recent years, computational systems biology has demonstrated its potential to contribute to a transformation in aging research, enabling a more systematic and quantitative view of aging [12].

**FIGURE 1:**
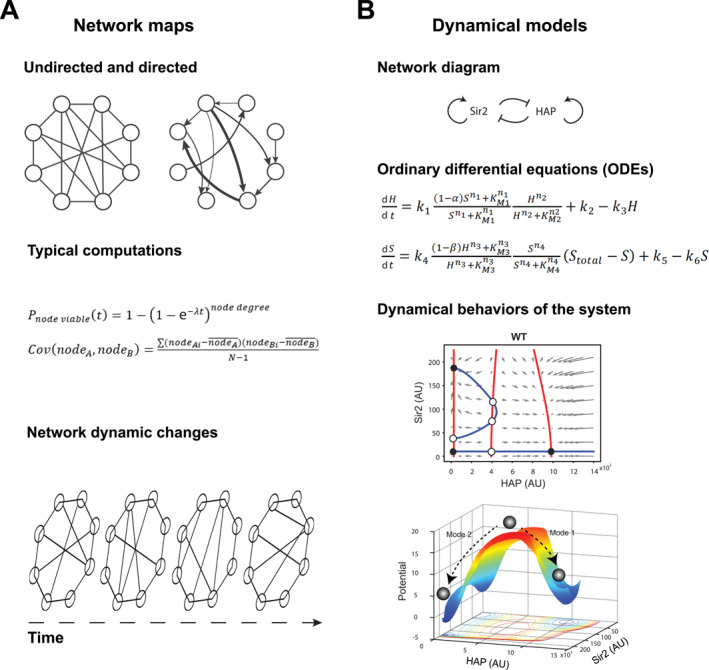
Schematic illustration of network map model and dynamical model. (A) Network map models involve large number of factors and are usually in the form of undirected or directed graphs to explore the connectivity and causal relations. Typical examples of computations used in network analysis include computing probability of node connections lost, as well as covariance between two nodes to determine directionality. Network maps can also be built and viewed to reflect time‐dependent changes. (B) Dynamical models focus on small systems with a few factors. Typically, ordinary differential equations are used to simulate the detailed kinetics and dynamic behaviors of the systems. Model simulation can be both deterministic and stochastic. Images in (B) were adapted and modified from Li et al. [21].

Systems‐level approaches used in aging studies can be generally categorized into two types that rely on different technologies and provide different perspectives of cellular aging. One type is referred to as network maps. Network maps are representations of biological networks, typically visualized as directed or undirected graphs composed of nodes representing factors such as genes or proteins [13]. Network maps can be assembled based on large‐scale datasets from systematic analyses of biomolecules (e.g., DNA, RNA, or protein expression levels or interactions) or correlations in molecular profiles. These maps have been very useful for determining the topologies of biological networks, such as transcriptional regulatory networks and protein interaction networks, and for characterizing functional biological pathways and processes [14, 15, 16]. In the context of aging, network mapping has been used to characterize age‐dependent changes in overall topology [17, 18] and stability [19, 20] of gene or protein networks. A drawback of this approach is that, although often genome‐wide in scale, network maps tend to be static, descriptive, and abstract (Figure 1A).

In contrast, dynamical models refer to the other type of systems‐level approaches. Compared to network maps, dynamical models are typically formulated around a particular set of components and interactions among them, and are devised to simulate the time‐dependent dynamics of these components [22, 23]. Dynamical models can be broadly divided into deterministic or stochastic. Deterministic models take the form of ordinary (temporal dynamics) or partial (spatiotemporal dynamics) differential equations for concentrations of components of interest (proteins, mRNA, and metabolites). On top of that, in order to account for stochasticity in biological systems, researchers apply stochastic models that are either based on stochastic differential equations for fluctuating concentrations or on methods that accelerate, via advanced algorithms and hardware utilization, direct simulation of stochastic biochemical reactions. In both cases, the models describe the dynamical behaviors of underlying systems. As a result, data derived from single‐cell time traces are particularly effective at refining model structures and constraining model parameters [24]. Dynamical models can confer quantitative and mechanistic insights into the operation and function of biological systems, and importantly, are often capable of accurately predicting the dynamic changes upon perturbations or interventions. Although this approach has only recently been introduced to aging research, it has already provided important insights into the cell‐to‐cell heterogeneity and regulatory mechanisms of aging processes. A drawback of this approach is that these models tend to be local, confined to a limited number of components, for which prior knowledge has been available (Figure 1B).

In this review, we discuss selected examples of network maps and dynamical models from recent studies of cellular aging. As an integration of network maps and dynamical models represents a challenging but rewarding goal in systems biology, we will discuss possible ways to connect these two approaches to achieve a more holistic and dynamic view of aging. Finally, we will discuss the questions and directions in aging research that may benefit the most from computational systems biology. We set out to provide a thorough and focused discussion of the approaches and the biological insights obtained from the recent progress in computational systems biology of cellular aging with a specific emphasis on some pioneering attempts of modeling the replicative aging of budding yeast *Saccharomyces cerevisiae*. For more comprehensive reviews of modeling organismal aging or systems biology of longevity in general, see Cohen et al. and Santiago et al. [12, 25]. Computational analyses of mammalian cell senescence have been extensively discussed in previous reviews [26, 27] and hence are not included in this paper.

## 2 NETWORK MAP MODELS IN CELL AGING STUDIES

In recent years, a number of studies have utilized network map models to probe aging. These range from abstract theoretical networks to empirical networks of yeast models, and to those probing networks in aging mammalian cells. The models are descriptive of gene and protein networks’ connectivity and topology, and show the capacity of inferring overarching theories and hidden causal relations that underlie cellular aging (Figure 1A).

Qin used network map models to evaluate the decline of connectivity, in other words the general stability of gene–gene interactions, of a cell’s overall gene network during aging [19]. In this study, the network map was highly abstract. Based on the simplified assumption that cell death occurs when any essential gene loses all connections with other factors, the author proposed an undirected graphic model, in which each node represents a gene, and nodes of essential genes are distinguished from those of nonessential genes. In addition, pertaining to the initial assumption, each essential node was assigned, on average, *n* connections with nonessential nodes but zero connection to other essential nodes. Considering the stochastic nature of gene–gene interactions, the author modeled gene interactions using a binomial distribution and postulated that the probabilities of interactions decline with time. Using the model, the author computed a time‐dependent probability of a certain essential node losing all connections, resulting in network failure and cell death. Besides the independent variable of time, the computation also incorporated parameters to represent the overall stability of gene interactions (the initial probability of interactions) and the overall configuration of gene networks (the number of interactions of an essential node). These parameters were adjusted to fit model simulation to experimentally determined lifespan curves from multiple aging‐related mutants. These mutants were found to affect the overall stability of gene interactions, rather than the overall network structure, to modulate lifespan. Another study by Vural et al. applied a different way to model network failure to show the independence of model fitting to survival curves on network topology [28]. Moreover, still using network models based on nodes and edges, they simulated the evolution of network structures and developed a random network as well as a scale‐free network, which preserved hubs of connections under different numbers of nodes. By assigning each node not only a failure rate but also a repair rate, such that node failure would not simply remove nodes from the networks, they demonstrated that both random and scale‐free network models reproduced survival curves of several model organisms. Although these models are abstractions of actual gene networks, and so cannot be directly compared with experiments, the overarching idea was insightful and theoretically sound, providing a general framework for considering the interplay between intracellular gene networks and cell aging.

In addition to such highly abstract models, network map models can also be empirical, reflecting specific genes to facilitate the extraction of mechanistic insights from large‐scale experimental datasets. Janssens et al. utilized a network map model to analyze age‐dependent transcriptomic and proteomic data and found that the uncoupling of transcript and protein levels of protein biogenesis related genes might statistically underlie many aging phenotypes [29]. They first collected transcriptomic and proteomic data from populations of aging yeast cells harvested at 12 different time points, corresponding to points in replicative lifespan. Analysis of the time series data revealed that genes related to translation regulation exhibited a significant decline of their transcript‐protein correlation during aging, in which the protein levels of the genes increased but mRNA levels decreased with age. The authors attempted to elucidate the role of these changes in aging using a network map model built upon the data they collected. In their directed graph model, each node represented the age‐dependent expression profile of a gene based on the transcriptome data, and an edge connected two nodes if they showed a significant partial correlation (the correlation between two genes when the influence of all other genes is removed). The direction of the edge was defined to represent the ability of one gene’s transcript profile to influence (predict) that of the other gene. The gene whose expression profile followed that of the other gene was called a “responsive” node, whereas the other gene was considered a “causal” node. The direction of the edge pointed from the causal node to the responsive node, indicating the potential causal relationship between the two nodes. All the nodes were clustered based on their interactions with other nodes. Interestingly, genes related to protein biogenesis were enriched in the node cluster with the highest causal ranking, which was based on the ratio of causal to responsive nodes in a cluster. Based on these results, the authors proposed that if age‐dependent changes of transcript and protein abundance occur in multiple sequential steps, then the change of protein biogenesis‐related genes is likely to precede and cause the expression changes of other genes. The network map model was used there to untangle the relationships of age‐induced expression changes among genes from transcriptomic and proteomic profile data. Although the conclusions from the analysis require further experimental validation, this study demonstrated a way to reveal potential cause–effect relationships in a plethora of interrelated factors, which could shed light onto mechanisms that drive the progression of aging processes.

Adopting the same age‐dependent transcriptomic and proteomic datasets, Guo et al. built another network model to explore the uncoupling between transcript and protein levels from a different angle [30]. They used known protein–protein interaction networks to determine pairs of interacting proteins, and deve‐loped protein interaction potential landscapes. Interaction potential of each pair of interacting proteins was computed using their transcript or protein abundances. If two interacting proteins both have large abundances, they were assumed to have high interaction potentials. Then “quasi‐potential” can be calculated as the negative logarithm of the ratio of an interaction potential in aged cells relative to that in young cells (the ground state). By plotting the quasi‐potentials, the authors were able to obtain interaction potential for all pairs of interacting proteins of interest at different ages relative to the ground state. For a landscape, *x* and *y* axes represented proteins sorted by different metrics, for example, effect on lifespan, node degree, etc. The *z* axis represented the calculated interaction potential (relative to the young state). Basins in the landscape reflected increased interaction probabilities, whereas ridges reflected lowered probabilities. They further defined essential versus nonessential factors based on their effects on lifespan (the deletion of an essential gene results in a lifespan of zero), and hub versus nonhub factors based on their number of interactions with other factors. They found that the age‐dependent landscapes generated using the proteomic data were different from the ones using the transcriptomic data. This uncoupling was especially striking for the interactions between essential proteins or for the interactions between hub proteins, although they seem to play different roles. Importantly, the authors discovered that the overlap of the two—the essential hub proteins—showed substantially increased abundances during aging, which represents a major contributing factor to the overall uncoupling of transcript and protein levels observed in aged yeast cells. This study, together with that of Janssens et al. described above, demonstrated how different computational approaches can be used to analyze the same dataset and obtain distinct but complementary insights to the mechanisms of aging. For example, the factors related to protein biogenesis identified by Janssens et al. may represent a primary functional category of the essential hub proteins in Guo et al., whereas some essential hub proteins from that study may suggest other driving factors of processes of aging, in addition to those related to protein biogenesis.

Similar approaches have also been used to study mammalian cell aging. Faisal and Milenkovic combined a protein–protein interaction network model and age‐dependent gene expression data to study the change of protein network topology during human aging [17]. They built a comprehensive protein–protein interaction network from well‐established protein databases and obtained human brain gene expression profiles from patients of various ages. Based on these data, the authors determined what proteins in the network were expressed at different ages, so that they could build a series of age‐dependent protein–protein interaction networks. The networks were visualized as undirected graphs, so both the global network topology and local topology (the number of connections around a specific protein) could be compared among networks corresponding to different ages. Interestingly, they did not observe any significant change of the global topology; instead, they identified many proteins whose age‐dependent expression changes led to substantial alterations in their local network topologies. Some of these proteins overlapped with previously known aging‐related factors, validating their analyses, whereas others might represent novel aging‐related targets to be tested. This study demonstrates how age‐dependent gene expression data could be integrated into network map models to achieve a more dynamic perspective of the overall system. Although the idea of “local network topology” is rather theoretical, such an analysis enabled a general visualization of how the complex network system changes with age and could facilitate data mining from large‐scale datasets to identify functionally important aging factors.

Besides discovering the driving force of cellular aging in a complex system, another important challenge is to identify the source of physiologic heterogeneity and lifespan variance within an isogenic population. Eder et al. recently presented an approach to explore this question in *Caenorhabditis elegans*, pertaining to the idea of modeling time‐dependent changes of the whole system [31]. This approach was enabled by *C. elegans* whole‐organism transcriptomics in isogenic populations at different ages obtained by Asynch‐seq. The authors used gene expression variances between isogenic worms as a measurement of nongenetic heterogeneity and found that, after dimension reduction, wildtype (WT) *C. elegans* showed variance in gene expressions not lying on the trajectory defined by age until very old age. They found that such nongenetic heterogeneity can be attributed to the decrease of correlation between mRNA contents in germline and somatic cells. Germline‐specific genes and somatic‐specific genes as two modules became more anticorrelated in aged individuals. The authors then demonstrated that knocking down of germline RNA polymerase II subunits not only reproduced the disparity between germline and somatic gene expressions but also recapitulated the nongenetic variance observed during the aging process. This could be shown by the observation that genes affected more by the knockdown, in both germline and somatic cells, has more weights in PC1—the axis along which the nongenetic variances lie in the above‐mentioned dimension reduction. Interestingly, germline ablation reduced both the nongenetic variance in gene expression profiles and the observed worm lifespan variance. After controlling the difference between germline and somatic mRNAs, the authors identified an additional 16 groups of co‐expressed genes, suggesting another 16 possible axes of nongenetic variance in the high dimensional space.

To generalize this strategy and find more causal factors of nongenetic variances in aged gene expression profiles, the authors sequenced transcriptomes of treated worms and assessed what treatments reca‐pitulated the age‐dependent nongenetic variances in the differential gene expression space. Using this approach, the authors identified 40 “hit” treatments, composed of knockdowns of functionally diverse genes forming 7 loosely co‐expressed clusters, but not including environmental perturbations such as tem‐perature or diet. Interestingly, in many of the knockdown screens they performed, if the target gene is more co‐expressed with another gene—an information extracted from interaction network models—the other gene would be affected more by the knockdown, showing that gene co‐expression data could serve as a good matrix when predicting the effects of a perturbation on the whole complex system. This recent study demonstrated the power of systems‐level analysis at identifying hidden regulatory factors, enabled by advances in sequencing technologies and bioinformatic analysis.

## 3 DYNAMIC MODELS IN CELL AGING STUDIES

Distinct from large‐scale network models described above, dynamical models focus on a limited number of factors and simulate their (spatio) temporal dynamics, as well as the kinetics of their interactions. This type of model can often be used to obtain mechanistic insights into the observed functional changes and generate predictions directly testable by perturbations in the systems (Figure 1B). A series of studies on gene expression noises in aging provides an interesting example. Initially, Acar et al. proposed that simple positive and negative feedback loops contained in gene expression control network had the capacity of network‐dosage compensation, meaning the invariance of gene expression level under the variance of gene copy numbers [32]. To further investigate the role of network‐dosage compensation in gene expression noise reduction, Peng et al. combined experiments and ordinary differential equation (ODE) modeling to understand compensated and noncompensated galactose (GAL) network subject to intrinsic stochasticity and extrinsic noises of gene expression in yeast [33]. They found that the system with compensation motif could better reduce the effects of extrinsic noise than the noncompensated system. Constrained by substantial single‐cell data, their dynamic model could nicely predict stochastic gene expression responses upon phy‐siological and genetic perturbations.

Successful modeling of the GAL regulatory system gave the authors important handles to study noise dynamics in aging cells [34]. Interestingly, they observed that gene expression noise in the GAL system decreased during aging until the late “catastrophe” phase (the final four generations of replicative lifespan). In order to explain this phenomenon, the authors applied their stochastic model of the GAL system and focused specifically on the role of promoter ON and OFF states transition rates. The model suggested that an age‐dependent parallel increase in both the ON and OFF rates of the promoter state transition could reduce the expression noise while keeping the expression level unchanged, recapitulating the experimental observations. These modeling results were consistent with an earlier study proposing that intrinsic gene expression noise was predominantly determined by stochasticity in promoter state transitions [35] and aligned with age‐dependent loss of chromatin stability observed previously [36].

This series of studies [32, 33, 34] demonstrated that dynamical models of a well‐established gene regulatory network, when combined with high‐resolution aging data, could shed light onto the novel effects of aging on cellular systems and the molecular mechanisms underlying these effects. In approaching a general aging‐related process, Erjavec and colleagues developed a dynamical model to study the role of asymmetrical segregation of damaged protein aggregates during cell aging [37]. The primary variable in the model corresponded to total damaged proteins in the cell, independently of any specific genes. The authors used ordinary differential equations to compute the rates of changes of intact protein and damaged protein concentrations, which are governed by protein production, degradation, and conversion of intact protein to damaged protein. Partitioning of intact and damaged proteins between a mother cell and its progeny was governed passively by asymmetrical cell division (uneven protein segregation depending on cell sizes) and actively by specific retention of damaged proteins in the mother cell. Damaged proteins could slow cell growth and eventually lead to cell senescence when their total level reached a threshold. By simulating the growth (fitness) of aging colonies, the authors found that both asymmetrical cell division and mother cell retention of damaged proteins could enhance the fitness of the population and delay clonal senescence. Interestingly, when combined with asymmetrical cell division, damage retention in mothers was only beneficial at high damage production rates, whereas a symmetrically dividing population could benefit from damage retention at all damage levels. These results highlighted an important role of damage retention mechanisms in symmetrically dividing cells, which led to the discovery of uneven damage partitioning in *Schizosaccharomyces pombe*. The detailed mechanisms underlying damage retention and segregation [38, 39, 40] were largely unknown when that study was conducted. Therefore, that model remained simple and abstract, without considering specific factors or regulatory networks. Because of that, it could be generally applied to the aging of various types of symmetrically dividing cells, such as *Escherichia coli* and mammalian epithelial cells.

More recently, Vedel et al. combined theory with experiments in bacteria and incorporated environmental stress into the model of damage partitioning during aging. They found that, on the population level, asymmetrical damage segregation could enable a healthy subpopulation that divided quickly and sustained the overall health of the colony under high stress conditions. At the same time, on the single‐cell level, the degree of asymmetrical segregation increased with stress level, further contributing to an adaptive effect [41]. Some additional modeling analyses have been conducted along this line of research [42, 43, 44, 45], which demonstrate the power of generalized dynamical models on advancing a fundamental understanding of aging biology.

Modeling the dynamics of general damage factors has also been used to guide the development of theories explaining organismal aging. In aging of many species, mortality rate increases exponentially with age, but the acceleration of such increase of mortality rate decreases among surviving old individuals. This is a phenomenon described by the Gompertz law [46]. One attempt was made to use simple dynamic modeling to understand the relation between mortality rate and damage on transcriptional stability [47]. Although many model organisms show increasing mortality rates that follow the Gompertz law, some organisms with an exceptional long lifespan, such as naked mole rats, show a constant mortality rate. Given that these long‐lived organisms feature stable gene expression profiles, the authors modeled the dynamics of damage rate of transcriptional regulation with an inter‐dependent two‐equation ODE model. The model is an abstraction of actual genes and proteins, and described the idea that instability in the genome or epigenome would cause alteration in the proteome. Moreover, since many proteins regulate gene expression, the altered proteome further influences the genes. The two ODE equations described the occurrences of “damaged genes” and “damaged proteins”, respectively. Terms in the equations that described production and degradation of the “damages” represented mutual influence between gene and proteins, external stresses, and also internal damage repair mechanisms. The authors showed that enhanced repair mechanism could account for transcriptional stability and mortality rates in naked mole rats.

By modeling the dynamics of senescent cell production and removal, Karin et al. also provided an explanation of the Gompertz law and demonstrated the generalizability of their model [48]. By defining time‐dependent mechanism and senescent cell abundance‐dependent mechanism for both production and removal of senescent cells, the authors generated 16 theoretically possible models based on whether each mechanism is present or absent. They scanned which model best recapitulated in vivo longitudinal tracking of senescent cell abundance in mice, by first writing all mechanisms into one equation with three terms—production, removal, and noise—and then setting every combination of parameters to 0, dictating the presence and absence of mechanisms during model simulation. The best‐fitting, simplest model was termed saturating removal model. It contained 4 parameters and described that senescent cells accumulate linearly with age, whereas their removal slows down by their own accumulation. The authors also showed that this model captured the half‐lives of chemically induced senescent cells in young versus old mice. By modeling the event of death as senescent cell abundance passing a threshold, the model further analytically reproduced the Gompertz law in mice and in human, with modest adjustments in parameter values. The model’s implication of mechanism is particularly interesting, as it describes that individuals with high senescent cell abundance has lower removal rate, whereas those with low senescent cell abundance maintains removal better, thus explaining the deceleration of mortality rate among old individuals. The saturating removal model is also generalizable to the accumulation of general risk factors. So long as a causing factor of aging has fast turnover rate that slows down by self‐accumulation, the model recapitulated the change of mortality rate when switching diet in *Drosophila*. On top of that, with a modified production rate, the model also recapitulated the scaling of *C. elegans* survival curves under different temperatures.

Indeed, the survival curve is typically considered the concerted output of aging of a complex system, it is thus a very important phenomenon to understand. Researchers have been trying to recapitulate the curve with understandable models to deduce key factors of aging, but there are potential pitfalls. For example, a Weibull model showed better fitting to yeast survival curves under a variety of genetic perturbations [49] than the Gompertz model. However, it has also been shown that Weibull model is inherently more tolerant to noises in experimental data, so a better fitting model may not be more biologically informative [50]. Such challenge is relevant to other dynamic modeling as well. Therefore, it is important to utilize iterations of experiments and modeling, focusing on testable predictions that can be used to evaluate and refine models based on multiple rounds of experimental perturbations.

The same saturating damage removal model, when coupled with single‐cell techniques to track damage dynamics in individual *E. coli* cells, further elucidated the mechanism behind damage accumulation and determination of cellular lifespan [51]. The authors utilized a microfluidic device and propidium iodide, which is a fluorescent dye that marks damaged cell membrane to track damage accumulation dynamics in single *E. coli* cells. They found that initial conditions including initial damage, cell size, and cell cycle phase did not explain the variance in cellular lifespans. Instead, a damage removal model improved and fine‐tuned with experimentally collected damage distribution among cells at different time points could reproduce the observed damage accumulation dynamics and lifespans. The model pointed to a mechanistic insight that the saturation of damage removal amplified the stochasticity in damage accumulation to eventually cause the variance in cellular lifespans. The authors also used the model to make more detailed predictions about damage dynamics upon interventions of damage production or removal. The single‐cell tracking technique allowed them to fully test and confirm the predictions in a mutant *E. coli* strain without a master regulator of stress response. This recent study not only confirmed the generalizability of the saturating removal model but also emphasized the importance of advances in cell tracking techniques in studies of aging dynamics.

Besides population level survival curves, single‐cell technology in yeast aging studies in conjunction with dynamic models have been used to understand some complicated phenomenon previously veiled by population‐level data. Traditional population‐level data suggested that many age‐related detrimental factors gradually accumulate throughout the lifespan, resulting in a gradual functional decline in aging cells. However, using a single‐cell imaging approach in budding yeast, Morlot et al. found that a discrete time point referred to as the senescence entry point (SEP) was obscured by population‐level data [52]. They discovered that for individual aging yeast cells, functional declines were largely initiated after SEP. Previous studies revealed that, during yeast aging, the ribosomal DNA (rDNA) region containing 100–200 copies of rDNA tandem repeats showed elevated rates of DNA double strand breaks and recombination, leading to the spontaneous formation of extrachromosomal rDNA circles (ERCs) excised from the rDNA region. ERCs are self‐replicating DNA circles, asymmetrically segregated to mother cells during cell divisions, resulting in its exponential accumulation in aging mother cells [53]. Morlot et al. proposed that the accumulation of ERCs over a certain threshold correlates with entry into cellular senescence. Their hypothesis was supported by observations that pre‐rRNA levels increased along with ERC accumulation, whereas levels of ribosomal biogenesis factors remained constant, resulting in a de‐synchronization within protein biogenesis machinery. Based on these data, the authors built a stochastic model to predict cellular lifespan based on molecular events related to ERC formation and accumulation. The model incorporated three steps: the stochastic excision of the first ERC from the rDNA region, the self‐replication of ERCs until reaching the SEP threshold, and the process between SEP and cell death. Although this model did not simulate detailed dynamics of molecular processes, it aptly reflected the stochastic nature of sequential events including the occurrence and accumulation of ERCs, and cell death after senescence. Moreover, in order to reproduce the data from *fob1Δ* cells, in which a major fraction of cells did not experience SEP, the authors added an ERC‐independent process to cell death in their model. Their model was able to reproduce the lifespan curves of WT and several well‐known aging mutants and supported a scenario for at least a subpopulation of cells, in which aging can be initiated by a purely stochastic event, defined as the excision of the first ERC from rDNA. Although the probability of this event can be age‐independent, the cumulative probability will inevitably increase with time, resulting in the unavoidable start of the aging clock driven by the subsequent exponential growth of ERC copies.

In addition to simulating well‐established systems, dynamical models can also be constructed to interpret and understand new experimental observations, which can lead to discovery of novel biological interactions. Recently, our group combined microfluidics with time‐lapse microscopy to track a large number of single yeast cells throughout their entire lifespans. We found that isogenic yeast cells diverged early in life toward two different paths of aging marked by distinct phenotypic changes such as daughter cell morphology and cell cycle length [36, 54]. During aging of individual cells, these paths were mutually exclusive and largely irreversible, suggesting the existence of a fate decision circuit that drives the divergence of aging paths. In theory, two mutually inhibitory factors can constitute a “toggle switch” circuit underlying such fate decision processes. To search for the molecular basis of the phenotypic divergence in aging and the potential “toggle switch,” we developed florescent reporters to monitor the dynamics of aging‐related molecular processes. We identified chromatin instability and mitochondrial biogenesis pathways, which mediated the two types of age‐induced phenotypic changes (Mode 1 aging vs. Mode 2 aging), respectively [21]. Furthermore, through genetic perturbation analyses, we found that the lysine deacetylase Sir2 and the heme‐activated protein complex (HAP), major regulators of the two pathways, inhibit each other, forming a toggle switch circuit. We devised deterministic and stochastic models of this circuit, which captured its multi‐stability nature (a system with multiple steady states) and could nicely reproduce the single‐cell aging trajectories in WT and various mutants. Importantly, the model predicted the emergence and enrichment of a third long‐lived aging path (a new longevity steady state of the system) upon overexpression of both Sir2 and HAP. We performed genetic engineering to test the prediction experimentally and indeed enriched this long‐lived population of cells, never observed before in WT cells, which led to a dramatically extended lifespan of the whole cell population.

The model of the core aging circuit further guided our pioneering attempt of using synthetic biology to engineer cellular aging processes [55]. The model showed that the endogenous Sir2‐HAP toggle switch mediates the commitment to detrimental steady states with prolonged rDNA instability or mitochondrial dysfunction, both of which could lead to cell death. Based on the insights from modeling, we hypothesized that rewiring the toggle switch into a negative feedback loop could create a genetic oscillator, which can prevent aging cells from commitment to either detrimental steady state and thereby lead to a longer lifespan. Under guidance of modeling, we replaced the native *SIR2* promoter with a *HAP*‐inducible *CYC1* promoter, enabling positive transcriptional regulation of SIR2 by *HAP*. Additionally, to allow Sir2 to inhibit *HAP* transcription, we inserted a construct containing the *HAP4* gene (which encodes a key HAP subunit) under a constitutive promoter into the rDNA region, which is subject to transcriptional silencing by Sir2. The resulting rewired strain exhibited oscillations in Sir2 levels, along with periodic cycles of rDNA silencing and heme biogenesis throughout aging. Importantly, the system did not result in a prolonged commitment to either rDNA silencing loss or heme depletion, leading to a remarkable 82% increase in yeast lifespan—a record for lifespan extension through genetic interventions. This work demonstrated, for the first time, a direct causal link between gene network architecture and cellular lifespan and highlighted the power of computational modeling in guiding rational design of gene circuits for longevity.

Our modeling approach can also advance our understanding in environmental control of aging [56]. Previous research has demonstrated that limiting glucose increases the activities of both Sir2 and HAP, which in turn can extend lifespan in yeast and other organisms. This raised the possibility that altering environmental conditions could create a longevity steady state similar to the one induced by genetic overexpression. To explore this idea, we examined the impact of varying glucose concentrations (from 5% to 0.02%) on yeast aging. We found that glucose levels modulate Sir2 and HAP activities in a dose‐dependent manner, influencing the balance between these two factors and ultimately guiding the aging process. Specifically, reducing glucose favored Mode 1 aging, which is characterized by rDNA silencing. It is worth noting that a glucose concentration of 0.1% resulted in a balanced, intermediate state for Sir2 and HAP, creating an optimal environment for lifespan extension. This intermediate balance appeared to establish a longevity steady state, maximizing lifespan compared to other glucose levels tested. Additionally, we demonstrated both theoretically and experimentally that periodic fluctuations in glucose levels could stabilize the system around this intermediate state of Sir2 and HAP activity, leading to lifespan extension without necessarily establishing a new longevity steady state.

Besides the knowledge gained about underlying gene regulatory circuits, rich cell morphology information was also collected in the tracking of single‐cell yeast aging. Our observation of two aging trajectories with distinct daughter cell morphologies—tubular elongated buds in Mode 1 aging versus spherical small buds in Mode 2 aging—facilitated the exploration of cell morphological control during the aging process, an important question in aging research. Tsai et al. [57] developed a combination of a 3D coarse‐grained particle‐based model that simulated the mechanical changes of the cell surface, and a reaction diffusion model that simulated the polarized spatial distribution of growth signals. The polarization of growth signals dictates the insertion region of new cell surface material, whereas changed 3D cell shape determines the further changes of signal polarization. The authors considered elongation of daughter cells in Mode 1 aging as a case of mis‐regulation of cell morphogenesis during the aging process. With model simulations, they found that this could happen when growth was restricted to the bud tip, whereas the addition of new surface material was faster than relaxation of surface. The tubular shape would be stabilized as the polarization of growth signal decreases at the end of a cell cycle. These simulations suggested potential mechanisms for future experimental validations.

Unlike many other modeling analyses that were built upon previously known networks, our modeling work on yeast aging was guided by the compelling experimental observations of single‐cell phenotypes and dynamics, without prior knowledge of the regulatory network. Hypothetic network structures underlying the observed single‐cell dynamics had been first conceived and had been used to guide our experimental identification of the molecular components and underlying mechanisms. The model generated testable predictions, leading to new biological insights and the possibilities for new pro‐longevity interventions in yeast and beyond. Our studies also highlighted the importance of the recent advances in measurement technologies, which enable tracking of single‐cell aging dynamics, invaluable for conceiving and constraining dynamical models. Reviews of single‐cell technologies used in aging studies can be found elsewhere [24, 58, 59].

## 4 INTEGRATING NETWORK MAPS AND DYNAMICAL MODELS

As demonstrated by the examples discussed above, network maps reveal the general architectures of large‐scale biological systems, whereas dynamical models describe the mechanistic operation of a particular set of regulatory factors. Network maps are powerful for characterizing the global topologies and functional relevance of molecular networks, but they may be static and descriptive; in contrast, dynamical models can advance understanding about the mechanistic causal connections among the network structure, dynamics, and function, but they tend to be local, thus limited by the scale. It will be rewarding to integrate these two types of computational analyses to empower a more comprehensive and dynamic view of cellular aging processes.

**FIGURE 2:**
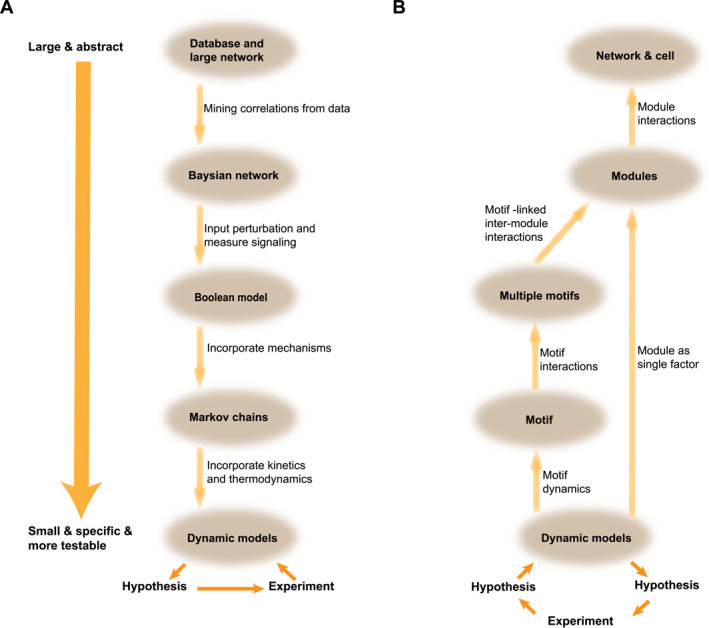
Schematic representation of the procedures for integrating network maps with dynamical models, with (A) the top‐down strategy, or (B) the bottom‐up strategy.

To address these challenges, a top‐down strategy has been depicted, based on the currently more prevalent bioinformatics methodologies [15] (Figure 2A). It suggests that starting from a “scaffold” of all interactions drawn from databases, a comprehensive network map can be built. Then experimental data can be used to derive smaller Bayesian networks of interest [60, 61]. Further perturbation data allow one to convert Bayesian network models into Boolean models that are capable of simulating an unrealistic, discrete step‐by‐step propagation of the perturbations throughout the network [62]. A different approach had been attempted to achieve the same goal by building sets of interacting genes that are relevant to certain perturbations and phenotypes [63]. Both approaches aim to identify more focused networks that are relevant to the perturbations or phenotypes of interest. Incorporation of kinetics to Boolean models can then enable construction of dynamical models [64]. In this way, dynamical models may be formulated to describe and simulate functionally specific subnetworks of large‐scale genome‐wide network maps.

We propose here the possibility of a bottom‐up strategy, starting with dynamical models of small network motifs, which can be further connected and expanded to simulate the dynamics of a larger network system (Figure 2B). A limitation of standard dynamical modeling is that this approach is most useful for networks with a small number of components. For large‐scale networks with many components, most dyna‐mical models are prone to overfitting, caused by large numbers of free parameters and the scarcity of experi‐mental data, which compromises the models’ ability to provide mechanistic insights and make meaningful predictions. Therefore, we propose instead to start with building dynamical models of small network motifs that can be well‐constrained by experimental data and then connect these network motifs together into a large network [65] by modeling the dynamic interactions among these motifs.

Network motifs were defined as particular patterns of interconnections among network components that occur significantly more often in real networks as compared to in random networks [66]. Based on this definition, efforts have been made to find transcriptional regulation network motifs and characterize their properties by ODE models [67]. Now, the functional roles of many motifs in cellular information processing are well understood. For example, negative feedback loops can mediate delayed refractoriness of information processing, such that the system can have a time window to respond to input signal before the negative feedback suppresses further activation by spurious signals [65, 68, 69]. Positive feedback loops can boost the response to later exposure to the same signal, and its underlying dynamics can function as a bistable switch, so that this motif drives genetically identical cells to one of the two steady states that respond differently to later stimulation [70, 71]. Feedforward loops can accelerate or delay the response time of gene expression based on their specific structures [72], and can contribute to information storage by triggering formation of mRNA granules during mRNA transcription, enabling a prolonged gene expression response to input signals [73].

Although the general properties of certain motifs are well studied, the exact dynamics of these motifs in the context of various biological systems will need to be further examined by dynamical models and constrained by experimental data. The interactions between components of motifs might include different types of regulation, such as protein–protein interaction and transcriptional control, thus operating with different time scales and kinetics [74]. This kind of complexity, as well as stochasticity in molecule interactions, is within the scope of dynamical modeling.

An understanding of the general dynamic properties of network motifs can also help us identify new interactions between functionally relevant factors based on experimental observations. For example, a bimodal response suggests the presence of a positive feedback loop or a mutual inhibition motif, whereas an oscillatory response might suggest the presence of a delayed negative feedback loop. In this way, instead of mining small motifs of interest out of larger networks, it is also possible to discover and model new interactions and new network motifs based on the observations of the system’s behaviors. Time trace experiments can be performed to constrain model parameters and refine model structures. Models will then enable investigators to generate predictions, which will be tested in further experiments. It is important to use iterations of experiments and modeling to improve and test the models’ performance and predictive power, both within the study of motif and in the pursuit of understanding of aging in general. A successful example of this approach in aging research is the recent analysis of the Sir2‐HAP circuit in yeast aging [21, 55, 56]. As described in the section above, through genetic perturbation experiments, we found that Sir2 and HAP form a “mutual inhibition” motif, which, in theory, could give rise to bistable fate decision responses. Our deterministic and stochastic models of this network motif helped us understand the mechanism underlying the divergence in single‐cell aging trajectories and generated predictions, which led to the creation of an engineered long‐lived mode of aging. In future studies, we can apply the same approach to other aging‐related small networks, such as the proteostasis network and the nutrient‐sensing pathways, and then quantify and model the interactions of these networks with the Sir2‐HAP circuit, with the goal of constructing, step‐by‐step, a comprehensive dynamical model of the aging process in yeast.

We propose that another angle of thinking about integration of the dynamical modeling approach with large‐scale networks can be based on the idea of “modularity” adopted from network map studies [75, 76, 77]. Network map analyses led to a hypothesis that gene networks were composed of modules of functionally related genes [78]. In support of this idea, studies found that genes emerging at closer points in evolution were more likely to interact with each other and evolution may have favored the emergence of clusters of genes that are subject to the same regulation and mediate the same function [79]. By approximating a gene module into a single variable, dynamical models can then be used to simulate the interactions among a limited number of gene modules in a large‐scale network, similar to modeling the interactions among individual genes or proteins in a network motif. The models can be constrained by experimental data focusing on the dynamics of the master regulator in each gene module, which represents the entire gene module. Similarly, dynamical models can also be applied to other types of interacting gene groups, clustered from expression profile datasets using dimensionality reduction methods, such as principal component analysis [80] and uniform manifold approximation and projection [81]. If the master regulators of gene modules are connected into specific topologies similar to those of network motifs, the models of network motif dynamics can be readily applied to simulating and understanding the dynamics of gene modules within a large‐scale network.

For aging research in particular, we anticipate that quantitative analyses of the interactions among network motifs or gene modules will provide important biological insights into the mechanisms of aging, as age‐dependent alterations of these interactions would most likely affect the overall topology and stability of the genome‐wide network [82]. For example, protein chaperones could be central mediators connecting many functional modules of protein networks via weak interactions. Breakage of the interactions around chaperones could lead to increasing noise and instability of the overall network during aging [83]. Dynamical models of such central mediators hold the promise of shedding light onto how aging influences the coordination among different network motifs or gene modules and affects the topology and function of gene networks, resulting in aging phenotypes and functional deterioration.

## 5 PERSPECTIVES

Cellular aging is highly complicated—not only due to complex dynamics of aging‐related factors but also due to intricate interconnections between these factors. New systems‐level approaches that integrate stochastic and nonlinear dynamical systems with large datasets are required to address such complexity. Above we have discussed the recent progress in computational modeling in aging biology and proposed potential strategies to integrate network maps with dynamical models to study comprehensive aging‐related networks. Below we list some directions or questions in the biology of aging where these approaches may be especially helpful.

### 5.1 Toward a comprehensive molecular network of aging

**FIGURE 3:**
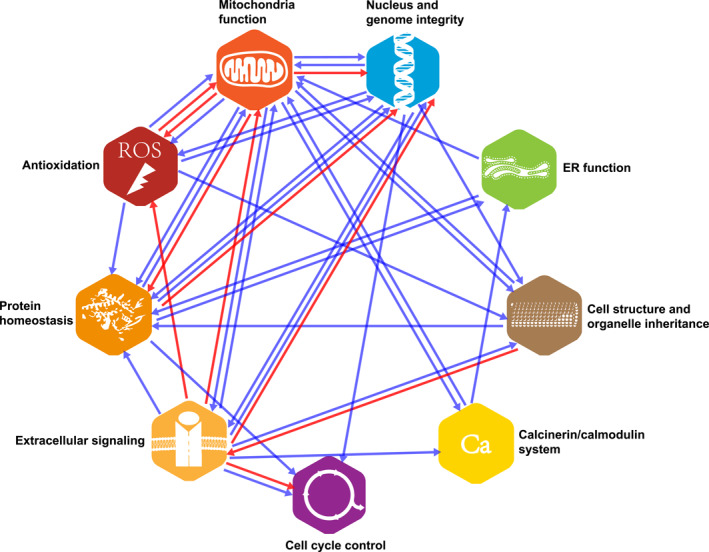
Schematic representation of known aging‐related interactions between functional components of a cell. A blue edge indicates an interaction between two components, which confers an anti‐aging effect under the normal physiological condition. A red edge indicates an interaction between two components, which confers an anti‐aging effect upon environmental perturbations, such as caloric restriction.

Many examples have been identified of cross‐talk among molecular and cellular hallmarks of aging [5] (Figure 3). However, understanding how these interactions operate dynamically to drive aging and how these hallmarks are temporally coordinated in individual aging cells remains elusive. New measurement technologies, in combination with dynamical modeling, can enable us to quantify the dynamics of specific interconnected aging hallmarks and understand the functional consequences of their interactions on cellular phenotypes and lifespan. For example, our analyses of the Sir2‐HAP circuit revealed the dynamic interactions between chromatin instability and energy metabolism and how these interactions underlie the single‐cell aging trajectories. Building upon this work, the next step would be to identify the interactions between other aging hallmarks and quantitatively analyze the dynamic coordination of these processes/pathways during aging. In fact, the latest studies from our group and others have uncovered new interactions between chromatin instability and loss of proteostasis [84, 85]. Based upon these findings, dynamical models can be developed for these newly defined interactions and combined with the existing Sir2‐HAP circuit models to form a larger‐scale model of aging. Systematic expansion along this line of research will eventually lead to a mechanistic, dynamical model of aging, which will be comprehensive and, at the same time, predictive because it will be well‐constrained by fine‐grained single‐cell data.

As discussed above, a complementary approach is to start with the existing datasets of age‐dependent gene expression profiles [29, 86] and group genes into modules based on their functions or using dimensionality reduction methods. Single‐cell time‐lapse measurements and dynamical modeling can then be applied to quantify the dynamic interactions among a limited number of representative genes for gene modules and evaluate their effects on aging phenotypes and dynamics. For example, Janssens et al. have grouped genes into multiple modules based on their expression profiles and proposed potential causal interactions among these modules [29]. Further studies can then focus on the dynamics of selected genes, which are representatives of these modules, in single aging cells. Genetic perturbation experiments can be performed to verify their interactions and functional relevance to aging. Based on these data, dynamical models can be constructed to simulate the dynamics of gene module networks and how these modules coordinate to shape aging trajectories in cell populations and in individual cells. These analyses, through a top‐down perspective, will also lead to a systems‐level dynamical model with predictive power at the single‐cell resolution.

### 5.2 Why do isogenic cells age differently?

Cellular aging is a highly heterogenous process wherein genetically identical cells can age with strikingly different phenotypes and at significantly different rates. However, the sources of such variability remain largely unknown. Although gene expression is intrinsically stochastic at the single‐cell level, whether and how these expression noises can underlie the sharply distinct aging phenotypes and lifespan in single cells remains unclear. Building upon the identified molecular networks of aging and single‐cell time trace data, stochastic simulations will provide important insights into how variations in the expression of specific aging‐related genes can be propagated through complex interactions within the aging‐related networks and contribute to the different phenotypes observed in isogenic aging cells. For example, a positive feedback loop or a mutual inhibition circuit can convert modest variations in gene expression into switch‐like bimodal responses, which can then lead to discrete phenotypic states. Several modeling studies have attempted to explore the variability in yeast aging and have made interesting findings regarding the stochastic behaviors of specific aging‐related factors or processes [21, 34, 52, 54]. Once the comprehensive molecular network of aging is constructed as described above, a systematic analysis can be performed to examine the cell‐to‐cell variability in all the major network components during aging. Experiments and models can be designed to evaluate the source and propagation of the expression variances and their contributions to the heterogeneity in aging phenotypes and dynamics.

### 5.3 How do environmental conditions shape cellular aging dynamics?

It has been shown that cellular lifespans can be substantially influenced by environmental conditions. For example, caloric restriction, or more generally mild stresses, can promote longevity from yeast to mammals and represents a robust approach to extend lifespan [87, 88, 89]. Recent studies revealed that the effects of environmental nutrient conditions are primarily mediated by highly conserved nutrient‐sensing kinases, such as the mechanistic target of rapamycin, AMP‐activated protein kinase, and protein kinase A [90, 91, 92]. These kinases regulate a wide range of metabolic and cellular processes through complex signaling and transcriptional networks. However, how these regulatory networks change during aging and how they mediate the effects of environmental conditions on aging and lifespan remains largely unclear. Dynamical models, in combination with time‐lapse measurements, can be used to quantify the age‐dependent dynamics of these networks and advance mechanistic understanding of the interplay between environmental and genetic factors and its role in determining aging dynamics. In particular, recently increased attention has focused on the benefits of periodic caloric restriction or intermittent fasting [93, 94, 95, 96, 97]. However, an exhaustive exploration of input dynamic patterns is not yet experimentally feasible given the complexity of a dynamic input. Systems‐level dynamical models will be able to sweep the variable space for dynamic inputs in silico and predict the relationships of the lifespan and dynamic input patterns with different amplitude, frequency, duration or interval. These predictions will guide further experimental testing of dynamic patterns of caloric restriction and will facilitate the design of dynamics‐based interventional strategies for promoting longevity.

Although still in its infancy, computational systems biology of aging is beginning to demonstrate its potential in pushing forward our understanding about the biology of aging from systems and dynamical perspectives. We envision that in the near future, an integration of network maps and dynamical models will enable the construction of the first ever comprehensive predictive model of aging networks, which will lead to new discoveries in the mechanisms of aging and new interventional approaches for extending the health span of living organisms.

## AUTHOR CONTRIBUTIONS

**Hetian Su**: Conceptualization; formal analysis; visualization; writing—original draft; writing—review and editing. **Nan Hao**: Conceptualization; funding acquisition; resources; supervision; writing—original draft; writing—review and editing.

## CONFLICT OF INTEREST STATEMENT

Nan Hao is one of the Editorial Board Members of *Quantitative Biology*. He was excluded from the peer‐review process and all editorial decisions related to the acceptance and publication of this article. Peer review was handled independently by other editors to minimize bias. The authors declare no conflicts of interests.

## ETHICS STATEMENT

This article does not contain any studies with human or animal subjects performed by any of the authors.

## Data Availability

This is a review article and does not contain original research data.
